# Disease Tolerance in *Toxoplasma* Infection

**DOI:** 10.3389/fcimb.2019.00185

**Published:** 2019-06-06

**Authors:** Stephanie J. Melchor, Sarah E. Ewald

**Affiliations:** Department of Microbiology, Immunology and Cancer Biology and the Carter Immunology Center, University of Virginia School of Medicine, Charlottesville, VA, United States

**Keywords:** tolerance, *Toxoplasma gondii*, resistance, cachexia, parasite, innate immunity, chronic infection

## Abstract

*Toxoplasma gondii* is a successful protozoan parasite that cycles between definitive felid hosts and a broad range of intermediate hosts, including rodents and humans. Within intermediate hosts, this obligate intracellular parasite invades the small intestine, inducing an inflammatory response. *Toxoplasma* infects infiltrating immune cells, using them to spread systemically and reach tissues amenable to chronic infection. An intact immune system is necessary to control life-long chronic infection. Chronic infection is characterized by formation of parasite cysts, which are necessary for survival through the gastrointestinal tract of the next host. Thus, *Toxoplasma* must evade sterilizing immunity, but still rely on the host's immune response for survival and transmission. To do this, *Toxoplasma* exploits a central cost-benefit tradeoff in immunity: the need to escalate inflammation for pathogen clearance vs. the need to limit inflammation-induced bystander damage. What are the consequences of sustained inflammation on host biology? Many studies have focused on aspects of the immune response that directly target *Toxoplasma* growth and survival, commonly referred to as “resistance mechanisms.” However, it is becoming clear that a parallel arm of the immune response has evolved to mitigate damage caused by the parasite directly (for example, egress-induced cell death) or bystander damage due to the inflammatory response (for example, reactive nitrogen species, degranulation). These so-called “disease tolerance” mechanisms promote tissue function and host survival without directly targeting the pathogen. Here we review changes to host metabolism, tissue structure, and immune function that point to disease tolerance mechanisms during *Toxoplasma* infection. We explore the impact tolerance programs have on the health of the host and parasite biology.

## Introduction: Disease Tolerance vs. Resistance

A successful immune response requires two distinct, but complementary components: “resistance mechanisms” and “tolerance mechanisms.” Resistance (or restriction) mechanisms directly target pathogens to limit microbial replication or dissemination. Examples of resistance mechanisms include antimicrobial peptides, complement, and degranulation by neutrophils or cytotoxic T cells. Tolerance mechanisms target host cell biology to improve tissue integrity and function in the setting of damage caused by the pathogen or the inflammatory response. Examples include extracellular matrix remodeling, the DNA damage response, antioxidant production, and shifts in cell metabolism (Ayres and Schneider, 2009, 2012; Medzhitov et al., 2012; Soares et al., 2014). Although tolerance mechanisms are often induced by and intimately related to an immune response, many disease tolerance programs are carried out by non-immune (non-hematopoietic) cell types. In fact, disease tolerance mechanisms were first described in plants, which lack a distinct cellular immune system, where they have been associated with tissue integrity, growth, and reproductive capacity (Caldwell et al., 1958).

Historically, immunologists have used the word “tolerance” to describe two processes that limit lymphocyte autoreactivity. In central tolerance, B or T lymphocytes with an affinity for self-antigens are deleted in the bone marrow or thymus before entering circulation. In peripheral tolerance, auto-reactive lymphocytes that arise later in development are deleted or rendered unresponsive to antigen (anergic) in peripheral tissues or in lymph nodes. Regulatory T cells (Tregs) play an important role in peripheral tolerance. Central and peripheral lymphocyte tolerance are included under the broader umbrella of so-called “disease tolerance” strategies because they are mechanisms of limiting inflammation and preventing auto-immunity but do not directly influence the pathogen. In this review, we refer to the broader definition of “disease tolerance” as mechanisms that support host fitness and survival during infection, not by directly targeting pathogen biology, but by shifting homeostasis to maintain tissue function during infection and inflammation (Martins et al., 2019).

**Figure 1:**
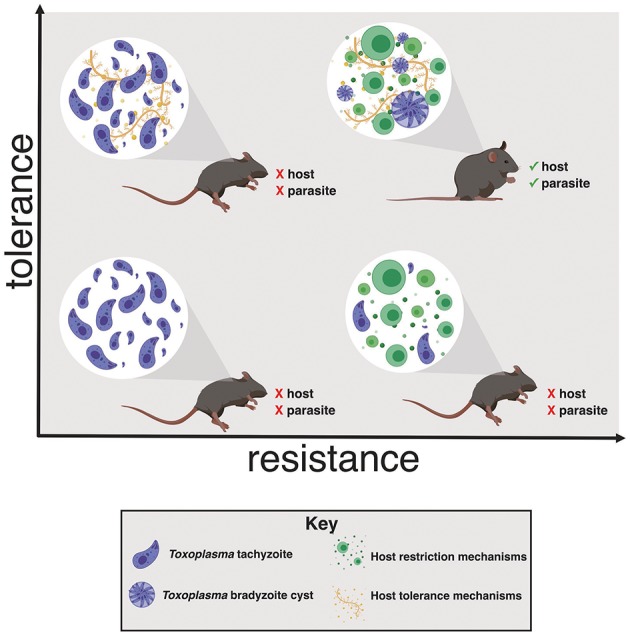
*Toxoplasma gondii* induces host tolerance and resistance mechanisms in successful infections. In the absence of a strong restrictive immune response the host succumbs to *Toxoplasma* overgrowth early in infection (left quadrants). Without tolerance mechanisms the host is susceptible to inflammation-induced pathology, even if parasite replication effectively restricted (lower right quadrant). Both resistance and tolerance mechanisms are necessary for host survival (upper right quadrant). This balance also benefits *Toxoplasma* by ensuring that the host survives long enough to enable bradyzoite cyst differentiation, a requirement for transmission to another host.

It is important to note that tolerance biology, like resistance mechanisms, represent a shift from normal homeostasis. The costs of overactive resistance mechanisms are well-established. Examples include bystander damage from reactive oxygen species, delayed wound healing, and recognition of autoantigens like DNA- or RNA-binding proteins. The negative effects of excessive tolerance programs are comparatively under-appreciated, but include mutations due to imperfect DNA repair, fibrotic wound healing and, potentially, metabolic disorders like Type II diabetes. We can assume that long-term reliance on tolerance mechanisms must be maladaptive, otherwise they would be selected for homeostatic function. This is an important consideration, as tolerance programs are receiving attention as therapeutic targets to interrupt the maladaptive consequences of acute inflammation (for example, sepsis or influenza infection) without increasing susceptibility to pathogens. Host survival during infection depends having both tolerance and resistance mechanisms, otherwise the host will succumb either to pathogen overgrowth or lethal immunopathology. As discussed in the following sections, it is also important to consider how promoting tolerance biology may benefit the life cycle of pathogens, particularly those that have evolved a strategy for long-term persistence within a host (Figure 1).

## Reinterpreting the Immune Response to *Toxoplasma* in the Framework of Disease Tolerance

*Toxoplasma gondii* is an obligate intracellular protozoan parasite that establishes life-long infection in a wide range of warm-blooded intermediate hosts, including rodents and humans. Three major haplotypes, types I–III, dominate Europe and North America which differ in virulence by several logs. Feline definitive hosts support *Toxoplasma* sexual recombination and shed millions of environmentally stable, highly infectious oocysts in feces. This confers a tremendous benefit for the parasite in terms of genetic diversity and dissemination potential, which suggests that rodents may be an important intermediate host for *Toxoplasma* given the predator-prey relationship between the two host organisms.

Intermediate hosts are infected by ingesting *Toxoplasma* oocysts or tissue cysts, the so-called bradyzoite form, from a previously infected intermediate host. *Toxoplasma* invades the small intestine, converting to the rapidly dividing tachyzoite form and inducing inflammation (Dubey et al., 1998). This process activates an immune response that is critical to the parasite's life cycle in two major ways. First, *Toxoplasma* infects and replicates within infiltrating immune cells (Courret et al., 2006). The parasite uses immune cells to disseminate systemically, reaching tissue sites that support chronic infection including the brain, muscle, and other tissues (Bierly et al., 2008). Second, chronic infection is defined by parasites shifting to a bradyzoite transcriptional program and clearance of most tachyzoites. This involves synthesis of a saccharide-rich parasite cyst wall which is necessary for the parasite to survive transit through the gastrointestinal tract of the next host (Bierly et al., 2008; Gregg et al., 2013). Thus, without a robust immune response, the parasite kills the host before this shift occurs and the opportunity for transmission is limited.

In the mouse, *Toxoplasma* is recognized by innate immune sensors including Toll-like receptors, and the NLRP1 and NLRP3 inflammasomes. Mice deficient in *tlr11* or its signaling adaptor *myd88* fail to control parasite replication after intraperitoneal (i.p.) infection (Yarovinsky et al., 2008). Similarly, mice lacking the inflammasome effector *caspases-1* and *-11* die of parasite overgrowth early in chronic infection (Ewald et al., 2014; Gorfu et al., 2014). Disabling the IFN-γ-regulated GTPase (IRG) system, comprised of GTPases which identify modified intracellular membranes and target them for degradation, leads to rapid parasite expansion and host death in inbred laboratory mice in a manner that depends on parasite genetics. Type II *Toxoplasma*, the most frequently isolated type in Europe and North America, expresses alleles of the effector proteins ROP5 and ROP18 which make it susceptible to IRG-mediated clearance. Hypervirulent type I and hypovirulent type III *Toxoplasma* express alleles of *rop5* and *rop18* that inactivate IRGs at the parasite vacuole improving intracellular replication in many inbred strains of mice (Reese et al., 2011; Etheridge et al., 2014). However, some wild-derived mouse strains are able to restrict growth of hypervirulent type I parasites in an IRG-dependent manner, suggesting that the IRG restriction pathway has imparted an important selective pressure on both host and parasite evolution (Lilue et al., 2013).

Innate immune recognition of *Toxoplasma* promotes IL-12 release, NK cell-mediated IFN-γ production and a Th1 polarized adaptive immune response (Sher et al., 1993; Hunter et al., 1994). The cellular mechanisms of these responses and the importance of CD8^+^ cytotoxic T cell response for host survival have been reviewed extensively elsewhere (Dupont et al., 2012; Sasai et al., 2018). Importantly, many cytokines and effector molecules central to this response have been shown to be necessary for parasite resistance. Specifically, mice deficient in IL-12 (Yap et al., 2000), IFN-γ (Suzuki et al., 1988; Deckert-Schlüter et al., 1996), IL-6 (Jebbari et al., 1998), TNF-α (Schlüter et al., 2003), iNOS (Scharton-Kersten et al., 1997), or their receptors die from parasite overgrowth in acute infection or early chronic infection. Host haplotype also plays a dominant role in parasite resistance. BALB/c mice are resistant to infection and express the H-2L^d^ MHC haplotype, which presents an immunodominant peptide from the dense granule protein GRA6 (Blanchard et al., 2008). In contrast, C57BL/6 mice have the H2b haplotype which presents ROP5, a lower abundance rhoptry protein that elicits a weak CD8^+^ T cell response, resulting in worse parasite restriction (Grover et al., 2014).

A number of immune cell-intrinsic pathways have been identified that mitigate Th1-mediated immunopathology during *Toxoplasma* infection which are relevant to disease tolerance (Table 1). At acute infection, mice deficient in IL-10 had a similar liver parasite burden as wildtype mice, but died from a cytokine storm of IL-12, TNF-α, and IFN-γ (Gazzinelli et al., 1996; Neyer et al., 1997). This phenotype could be reversed by depleting CD4+ T cells (Gazzinelli et al., 1996), or by depleting IFN-γ (Suzuki et al., 2000). More recently, Jankovic et al. showed that T-bet^+^Foxp3^−^ Th1 cells were a major source of IL-10 during chronic i.p. infection with 20 ME49 cysts. These IL-10-producing cells were required to limit fatal immunopathology during both acute and chronic infection (Jankovic et al., 2007). In 2005, Wilson et al. found that lethal *Toxoplasma* encephalitis in chronically infected IL-10^−/−^ mice is not due to higher parasite loads, implicating IL-10 as a tolerance effector in the central nervous system (CNS) during *Toxoplasma* infection (Wilson et al., 2005). Similarly, mice lacking IL-4 were more susceptible to *Toxoplasma* infection despite having slightly reduced cyst burdens and microglial nodules in the brain at chronic infection (Roberts et al., 1996). These data suggest that tolerance mechanisms induced during infection are critical to the survival of host. Although the mechanistic basis for the effect of IL-4 is currently unclear, it has been well-studied in other systems. One example is the rodent helminth *Nippostrongylus brasiliensis*. *N. brasiliensis* burrows through the skin then migrates to the lung where it is coughed up and swallowed to complete its life cycle in the gastrointestinal tract. *N. brasiliensis* chitin promotes IL-4, driving alveolar macrophages to limit lung pathology by producing insulin-like growth factor 1, resistin-like molecule-α and arginase-1 (Chen et al., 2012). These effectors dampen inflammation and promote the fibroblast wound repair response (Knipper et al., 2015). Importantly, the parasite's egg-laying stage occurs in the gut, after trans-lung migration. There may be significant selective pressure for *N. brasiliensis* to activate tolerance programs that promote wound healing and host survival so that the parasite has sufficient time for transmission.

**Table 1:** Summary of literature describing immunoregulatory pathways during *Toxoplasma gondii* infection.

**Observation**	**Reference**
IL-10^−/−^ mice die of acute encephalitis during *Toxoplasma* infection, associated with elevated cytokine levels, but not with enhanced parasite proliferation	Gazzinelli et al., 1996
IL-4^−/−^ mice succumb during acute *Toxoplasma* infection despite having fewer circulating parasites, but chronic IL-4 suppresses the restriction response, leading to increased pathogen burden and pathology	Roberts et al., 1996
Mice deficient in 5-lipoxygenase (the enzyme responsible for generating lipoxin A_4_) succumb to otherwise sublethal doses of *Toxoplasma*, have elevated circulating IL-12, IFN-y, and TNF-a, and have increased T cell infiltration, despite harboring lower cyst burdens than WT mice	Aliberti et al., 2002
Mice lacking the IL-27 receptor have enhanced mortality and CD4^+^ mediated tissue damage, independently of parasite burden during oral *Toxoplasma* infection	Villarino et al., 2003
Chronic IL-10 is required to limit fatal immunopathology mediated by CD4^+^ T cells in the brain during *Toxoplasma* infection	Wilson et al., 2005
IL-10 is required to limit fatal immunopathology at acute and chronic *Toxoplasma* infection. In this context, it is primarily produced by T-bet^+^Foxp3^−^ Th1 cells	Jankovic et al., 2007
IL-27 signaling promotes a subset of Tregs that limit intestinal pathology and improve host survival during oral *Toxoplasma* infection	Hall et al., 2012
Lamina propria-infiltrating Ly6C^hi^ monocytes release IL-10 and PGE_2_ that limit neutrophil-induced tissue pathology in oral *Toxoplasma* infection	Grainger et al., 2013
Gut infiltrating neutrophils create multicellular casts to prevent bacterial translocation, improving host survival independently of parasite burden	Molloy et al., 2013

In addition to their well-characterized roles in pathogen clearance and adaptive immune response activation during *Toxoplasma* infection, innate immune cells also play a critical role in mitigating tissue pathology. A 2013 study from Yasmine Belkaid's laboratory found that lamina propria-infiltrating neutrophils generated “casts” containing cells and extracellular DNA over regions of small intestine damaged by per oral (p.o.) *Toxoplasma* infection. These casts which appeared to limited bacterial translocation into host circulation. Depleting neutrophils with an α-Gr-1 antibody increased mortality without increasing parasite burden, indicating that the immune-commensal axis also plays an important role in disease tolerance during *Toxoplasma* infection (Molloy et al., 2013). During p.o. *Toxoplasma* infection, lamina propria-infiltrating Ly6C^hi^ monocytes were also shown to release IL-10 and prostaglandin E_2_ (PGE_2_) which limited neutrophil-induced pathology. Administration of a PGE_2_ analog during infection was sufficient to reduce intestinal tissue pathology and immune infiltration in the absence of monocytes without affecting parasite burden (Grainger et al., 2013).

T cells are important players in disease tolerance mechanisms during *Toxoplasma* infection. 5-lipoxygenase is the rate-limiting enzyme in the synthesis of lipoxin A_4_, an eicosanoid mediator of the resolution phase of an inflammatory response (Colgan et al., 1993). The importance of this pathway in disease tolerance is underscored by the observation that 5-lipoxygenase deficient mice succumbed to i.p. infection with 20 ME49 cysts by 35 days post-infection, despite harboring significantly fewer brain cysts than wild type mice. Death was associated with encephalitis, increased T cell infiltration into the brain and elevated circulating IL-12, IFN-γ, and TNF-α (Aliberti et al., 2002). IL-27 is part of the IL-12 family of JAK-STAT signaling cytokines with an emerging role in tolerance to *Toxoplasma* infection. WSX-1 knockout mice (which lack the IL-27 receptor) i.p. infected with 20 ME49 cysts succumbed by 13 days post-infection. No change in parasite burden was detected between knockout and wild type mice in peritoneal lavage fluid at 7 days post-infection, but knockout mice had elevated circulating IL-12, IFN-γ, increased inflammation and necrosis in the liver and lungs, and hyperactive and hyperproliferative CD4^+^ T cells in the spleen. Survival was partially rescued when CD4^+^ T cells were depleted (Villarino et al., 2003). Subsequently, the same group found that IL-27 limited production of IL-2 by activated CD4^+^ T cells, implicating IL-27 receptor as a negative regulator of T cell responses during *Toxoplasma* infection (Villarino et al., 2006). This conclusion was also supported by a 2012 study from the Hunter Laboratory where mice were p.o. infected with 100 ME49 cysts or i.p. infected with 20 cysts. IL-27 promoted development of Tregs at primary sites of infection, the small intestine or peritoneal cavity, respectively. IL-27^−/−^ mice succumbed to acute infection, which could be partially rescued by adoptive transfer of Treg cells (Hall et al., 2012). Although this result is consistent with a tolerance strategy, parasite burden was not directly measured.

IL-2 is also important for Treg biology in *Toxoplasma* infection. Using p.o. infection, several groups have shown a transient, but significant loss of Treg function in the small intestine (Oldenhove et al., 2009; Benson et al., 2012). Addition of IL-2 promoted Treg survival and prevented liver pathology, consistent with a role in disease tolerance in the liver. However, IL-2 treatment also resulted in a higher brain cyst burden (Oldenhove et al., 2009). Similarly, Benson et al. found that IL-2 treated mice had more Tregs and significantly higher brain cyst burdens causing lethality (Benson et al., 2012). Whether the inability to restrict cerebral *Toxoplasma* infection in these studies was due to Treg-mediated suppression of effector T cells or a direct effect of IL-2 on naïve or effector T cell activity is unclear. However, directly targeting effector T cell responses has been shown to reduce tissue damage in a mouse model of ocular toxoplasmosis. Specifically, in the retina both infiltrating antigen-presenting cells (MHCII expressing) and tissue resident retinal cells expressed PD-L1, the ligand for the T cell inhibitory receptor PD-1. PD-L1 expressing retinal cells were able to suppress splenic T cell proliferation *ex vivo* using a co-culture system with *Toxoplasma*-antigen loaded dendritic cells (Charles et al., 2010). These results suggest that retinal cells may be able to moderate local immune responses and reduce tissue damage by directly suppressing T cell activation in the eye. Cumulatively, these studies underscore the importance of targeting the T cell response to limit tissue damage and restrict parasite growth.

## Host Metabolic Dysregulation in *Toxoplasma* Infection

There is a growing appreciation that immune responses are intimately linked with shifts in metabolic homeostasis. This is a critical arm of pathogen resistance, for example, T cell activation requires a glycolytic burst; anorexia during infection mobilizes glycogen and lipid stores to support gluconeogenesis for the immune system; metabolic shifts are also used to sequester trace metals or nutrients to limit pathogen growth, often referred to as nutritional immunity (Hood and Skaar, 2012; Núñez et al., 2018). Shifts in nutrient utilization are also important mediators of disease tolerance. For example, diet restriction improves fruit fly survival during *S. typhimurium* infection without affecting bacterial load (Ayres and Schneider, 2009).

There is a growing body of literature showing metabolic shifts in the host during acute *Toxoplasma* infection. However, a role for these shifts in disease tolerance has not been addressed specifically. Using Swiss-Webster mice infected p.o. with 8 cysts of the human-derived Type II strain BGD-1, one group reported reduced circulating cholesterol and HDL at 14 days post infection (Milovanović et al., 2017). Untargeted proteomic analysis of sera isolated from BALB/c mice infected per orally with 10 Type II Pru cysts showed increased circulating amino acids and reduced choline levels in the first 21 days of infection (Zhou et al., 2016). The same group observed an increase in choline-derived phosphatidylcholine and phosphatidylethanolamine in the brains of infected mice (Zhou et al., 2015). Choline is an essential dietary nutrient as the precursor of phosphatidylcholine and the neurotransmitter acetylcholine. Phosphatidylcholine is a major component of cell membranes and is necessary for the packaging and export of very-low-density lipoproteins (VLDL) from the liver (Corbin and Zeisel, 2012). Together, these studies suggest that *Toxoplasma* infected mice shift toward amino acid and fat metabolism and away from glycolytic metabolism during *Toxoplasma* infection. This is consistent with a well-described, although transient, period of anorexia during acute infection in *Toxoplasma* infected mice which would induce such programs (Arsenijevic et al., 1997; Weiss and Dubey, 2009; Jin et al., 2017; Hatter et al., 2018). In mice infected with *L. monocytogenes*, anorexia-induced ketogenesis protected tissues from oxidative stress, whereas glucose supplementation increased mortality. Interestingly, the opposite effect was observed with influenza infection, suggesting that the effectiveness of tolerance programs, like restriction mechanims, depend on the pathogen (Wang et al., 2016). Similar experiments to look at the role of glycolytic and beta-oxidative host metabolism in *Toxoplasma* will be necessary to determine if anorexia respresents a host tolerance strategy or if the parasite capatilizes on altered host metabolism for infection.

It is well-established that scavenging host lipids is necessary for *Toxoplasma* survival *in vivo* and *in vitro*. For example, most of the cholesterol in *Toxoplasma* is derived from scavenged host LDL (Bisanz et al., 2006). Metabolic labeling with ^14^C-acetate showed that *Toxoplasma* must scavenge fatty acid precursors from its host to synthesize its full range of lipids (Sehgal et al., 2005). Recent studies indicate that *Toxoplasma* competes with the host cell for lipids at the level of lipid droplet recruitment, mitochondrial interaction, and vesicular transport for intracellular survival (Hu et al., 2017; Nolan et al., 2017; Pernas et al., 2018). Further studies are warranted to determine whether host metabolic shifts that mobilize lipid stores at a systemic (rather than a cellular) level benefit *Toxoplasma*. By contrast, there is growing evidence that altered metabolism has a long-term negative impact on the host during *Toxoplasma* infection. A series of studies by Arsenijevic et al. demonstrated that mice orally infected with 10 Me49 cysts undergo anorexia-associated hypermetabolism during acute infection. Nearly half of the infected cohort could not regain weight as they progressed to chronic infection. These “non-gainers” harbored a higher parasite load than mice that regained weight and sustained the hypermetabolic phenotype along with elevated circulating inflammatory cytokines (Arsenijevic et al., 1997, 2001). A subsequent study showed that non-gainers challenged with LPS had a more severe inflammatory response, worse pathology, and a longer rebound period than infected mice that gained weight (Arsenijevic et al., 1998). Related, a study by Kugler et al. showed that *Toxoplasma* infection led to long-term defects in thymus and lymph node structure, hindering naïve T cell responses to subsequent viral challenge (Kugler et al., 2016). More recently, the Wohlfert lab showed that oral infection with 5 Me49 cysts causes acute weight loss in mice and inability to regain weight as chronic infection progresses (Jin et al., 2017). Surprisingly, muscle inflammation and myositis were driven by Tregs, a population classically associated with tolerance, suggesting that sustained tolerance programs may negatively impact the host. In 2018 Hatter et al. similarly reported that C57BL/6J mice orally infected with Me49 cysts develop chronic cachexia defined by lean muscle and fat wasting and chronic elevation of circulating innate cytokines. Although cachectic mice recovered from acute ileitis, dysbiosis in the intestinal commensal microbiota population was sustained (Hatter et al., 2018). Loss of total gut commensal diversity and a shift toward “pathobiotic” Gram negative species is well-established in acute *Toxoplasma* infection (Heimesaat et al., 2006; Benson et al., 2009; Molloy et al., 2013; Hatter et al., 2018). Although the precise composition of outgrowth species is animal colony-dependent, several groups have reported *E. coli* outgrowth (Benson et al., 2009; Raetz et al., 2012; Molloy et al., 2013; Fonseca et al., 2015). This is interesting in the context of the Zhao Lab metabolomics data because these bacteria are major metabolizers of the choline derivative ethanolamine (Garsin, 2012). It is highly plausible that shifts in commensals influence nutrient availability, in addition to playing a better-described role in influencing host immunity during *Toxoplasma* infection

*Toxoplasma* interactions with major organs involved in nutrient regulation may have important implications for host metabolism as well. The liver coordinates dietary nutrient uptake (bile acid recycling), availability and storage (fat and glycogen), and detoxifies the blood. Liver resident macrophages called Kupffer cells screen incoming blood for pathogens and intestinal microbes that leak from the gut during intestinal inflammation, including during *Toxoplasma* infection (Molloy et al., 2013). *Toxoplasma* has been detected in the livers of mice at acute infection using bioluminescence, histology, and PCR in a number of studies using Type II strains (Silva et al., 2002; Di Cristina et al., 2008; Zhou et al., 2015; He et al., 2016a,b). In the first week of infection, *Toxoplasma* has been observed replicating in hepatocytes near regions of inflammatory infiltrate and focal necrosis in both Swiss-Webster and BALB/c mice (Atmaca et al., 2013; Bottari et al., 2014). Interestingly, Atmaca et al. also reported an expansion of hepatic stellate cells during *Toxoplasma* infection (Atmaca et al., 2013). During inflammation, hepatic stellate cells differentiate into myofibroblasts and produce extracellular matrix which is consistent with the induction of a tissue remodeling tolerance program during *Toxoplasma* infection. However, an important caveat with these studies is that they were performed with lethal doses of the hypervirulent Type I strain RH raising the question of relevance to hepatic infection with strains that are more commonly found in mice or humans. Using clinically isolated Type II strains, *Toxoplasma* cysts were found in the livers of infected Swiss-Webster mice as late as 33 weeks post-infection, indicating that the liver may be a reservoir for chronic infection (Autier et al., 2018). This is consistent with clinical reports of *Toxoplasma*-negative transplant recipients who have developed toxoplasmosis after receiving livers from sera positive donors (Assi et al., 2007; Galván-Ramírez et al., 2018). While there are no studies directly implicating *Toxoplasma* in the development of liver disease, approximately 30% of patients with chronic liver disease test sera-positive for *Toxoplasma* B1 compared to 6% in control populations (El-Sayed et al., 2016). These *Toxoplasma*-infected patients had significantly elevated circulating ALT and AST, clinical markers of liver damage, compared to uninfected patients with liver disease. Although these data do not imply causality, they are consistent with the interpretation that chronic *Toxoplasma* infection may negatively impact host fitness in diseases of co-occurrence.

*Toxoplasma* infection may also change the metabolic landscape of the liver. In a recent liver proteomics study, BALB/c mice intraperitoneally infected with 200 type II PYS tachyzoites had reduced signatures of fatty-acid oxidation proteins and an upregulation of cell death, inflammatory, and stress response pathways at 6 days post-infection (He et al., 2016b). A parallel liver transcriptomics study reported down-regulation of gene families involved in lipid metabolism, cholesterol and bile synthesis, and amino acid metabolism, with an increase in inflammatory transcripts (He et al., 2016a). Together, these studies provide evidence that *Toxoplasma* occupies a liver niche in acute and chronic infection and may directly contribute to shifts in liver metabolic homeostasis.

Adipose tissue depots are another important site of calorie storage and immune regulation. Fat tissues have long been described as an anti-inflammatory environment that become inflamed in diseases of the adipose tissue, including obesity and diabetes. As better tools have become available to survey low pathogen loads in tissue, researchers have begun to appreciate the frequency of microbial translocation to and persistence in this nutrient-rich environment. *M. tuberculosis, T. brucei*, and facultative pathogen strains of *E. coli* have been detected in this niche, indicating that it serves as a reservoir of infection for many pathogens (Neyrolles et al., 2006; Schieber et al., 2015; Trindade et al., 2016). More recently, *Toxoplasma* has been reported in the visceral fat following intraperitoneal infection and oral infection by bioluminescence assay and PCR (Di Cristina et al., 2008). Interestingly, using a stage-specific luciferase reporter system, di Cristina et al. showed that parasites in visceral fat expressed GFP driven by the SRS9 promoter, a gene product enriched during bradyzoite differentiation (Di Cristina et al., 2008). Future studies will be necessary to determine if fat is a long-term reservoir for *Toxoplasma*, and whether colonization of the fat is related to host metabolic shifts.

## Conclusions

*Toxoplasma gondii* has evolved sophisticated mechanisms to evade sterilizing immunity, yet activating a robust immune response is necessary to ensure host survival long enough for *Toxoplasma* encystation and transmission (Bohne et al., 1993, 1994). Disease tolerance programs are adaptations to cell biology and metabolism that allow tissues to function in the harsh environment of an inflammatory response (Tzelepis et al., 2018). However, tolerance adaptations must come at a cost to the host, otherwise they would be selected for homeostatic use. The literature reviewed here are consistent with a model where tolerance programs initiated in acute *Toxoplasma* infection, including immune-microbiota interactions, T cell-mediated responses and metabolic shifts fail to return to homeostasis in chronic infection. Emerging studies suggest that these shifts in homeostasis have sustained negative consequences for the host, including muscle wasting, and impaired responses to secondary immune stimuli (Arsenijevic et al., 1997; Kugler et al., 2016; Jin et al., 2017; Hatter et al., 2018). Whether or not these shifts in homeostasis confer a benefit to the parasite is an open question. Compromising rodent fitness in these ways would likely enhance the opportunity for predation by felines, the parasite's definitive host. Passage through a cat is extremely advantageous for *Toxoplasma* because feline hosts facilitate genetic diversity and range expansion. In this way, *Toxoplasma* may benefit from promoting tolerance programs that ensure host survival during the acute phase of infection, during the tachyzoite to bradyzoite transition, but ultimately impair host fitness in the long term.

## Author Contributions

All authors listed have made a substantial, direct and intellectual contribution to the work, and approved it for publication.

### Conflict of Interest Statement

The authors declare that the research was conducted in the absence of any commercial or financial relationships that could be construed as a potential conflict of interest.
